# Interferon induced protein 35 exacerbates H5N1 influenza disease through the expression of IL-12p40 homodimer

**DOI:** 10.1371/journal.ppat.1007001

**Published:** 2018-04-26

**Authors:** Anshu P. Gounder, Christine C. Yokoyama, Nicholas N. Jarjour, Traci L. Bricker, Brian T. Edelson, Adrianus C. M. Boon

**Affiliations:** 1 Department of Internal Medicine, Washington University in Saint Louis School of Medicine, St. Louis, MO, United States of America; 2 Department of Molecular Microbiology, Washington University in Saint Louis School of Medicine, St. Louis, MO, United States of America; 3 Department of Pathology and Immunology, Washington University in Saint Louis School of Medicine, St. Louis, MO, United States of America; St. Jude Children's Research Hospital, UNITED STATES

## Abstract

Pro-inflammatory cytokinemia is a hallmark of highly pathogenic H5N1 influenza virus (IAV) disease yet little is known about the role of host proteins in modulating a pathogenic innate immune response. The host Interferon Induced Protein 35 (Ifi35) has been implicated in increased susceptibility to H5N1-IAV infection. Here, we show that Ifi35 deficiency leads to reduced morbidity in mouse models of highly pathogenic H5N1- and pandemic H1N1-IAV infection. Reduced weight loss in *Ifi35*^*-/-*^ mice following H5N1-IAV challenge was associated with reduced cellular infiltration and decreased production of specific cytokines and chemokines including IL-12p40. Expression of *Ifi35* by the hematopoietic cell compartment in bone-marrow chimeric mice contributed to increased immune cell recruitment and IL-12p40 production. In addition, *Ifi35* deficient primary macrophages produce less IL-12p40 following TLR-3, TLR-4, and TLR-7 stimulation *in vitro*. Decreased levels of IL-12p40 and its homodimer, IL-12p80, were found in bronchoalveolar lavage fluid of H5N1-IAV infected *Ifi35* deficient mice. Specific antibody blockade of IL-12p80 ameliorated weight loss and reduced cellular infiltration following H5N1-IAV infection in wild-type mice; suggesting that increased levels of IL-12p80 alters the immune response to promote inflammation and IAV disease. These data establish a role for Ifi35 in modulating cytokine production and exacerbating inflammation during IAV infection.

## Author summary

Highly pathogenic influenza A viruses (IAV) are an important human pathogen that cause high mortality and can acquire the ability to cause pandemics. Following highly pathogenic H5N1-IAV infection, exaggerated inflammatory responses are detrimental to the host and lead to more disease; tipping the balance between protection and pathology. Understanding the role of host genes that enhance inflammation will lead to the identification of therapeutic targets and treatments to help lessen severe disease. Here, we report that the deletion of an interferon induced gene, *Ifi35* (interferon induced protein 35), in mice protects the host from severe morbidity following H5N1 infection. Ifi35 enhances inflammation following H5N1 infection by increasing pro-inflammatory cytokine production; notably, the cytokine IL-12p40 and its homodimer, IL-12p80. Blocking IL-12p80 in mice led to reduced weight loss following H5N1 infection. Thus, our results provide insights into the development of therapeutic agents against host factors, Ifi35 and IL-12p80, to help control inflammation and inflammatory disease states.

## Introduction

Infections with highly pathogenic IAV, such as H5N1-IAV, are characterized by high viral titers, excessive inflammation, and increased mortality after infection [1–3]; (WHO/GIP, 2017). While the host innate immune response is important for limiting IAV replication and disease; an aberrant innate immune response leads to overt cytokine production, immune cell infiltration, tissue damage, and more severe disease [4–8].

The relative contributions of virus replication and immunopathology in driving severe disease remain unclear and have fundamental implications in treating H5N1-IAV infections. Increased levels of the cytokines IL-6, IL-10, and IL-12 as well as the chemokines CXCL10 and CXCL2 have been reported to contribute to immune dysregulation and severe disease following H5N1 infection in humans and mice [9–12]. The production of several pro-inflammatory cytokines and chemokines are known to be amplified by type I interferon (IFN) receptor signaling. Several studies have suggested pathogenic roles for IFN and IFN-induced host proteins during IAV infection [13–16]. Thus, modulation of cytokine production and IFN signaling needs to be balanced to control virus infection but not promote excessive inflammation and immunopathology. Yet, the role of host proteins in regulating this balance of innate immune responses to IAV infection is not well characterized.

The host molecule Interferon Induced Protein 35 (Ifi35) has been implicated in increased susceptibility to H5N1-IAV in a genome-wide analysis of IAV-resistant and susceptible mouse strains [17]. Ifi35 has also been reported to interact with various host and viral proteins to modulate infection, innate immune, and inflammatory responses [18–28]. Until recently, all functional studies on Ifi35 have been done in tissue culture models but its role and activity *in vivo* after infection with IAV or other pathogens is not well characterized.

Here, we define a role for Ifi35 in IAV pathogenesis using an *Ifi35*^*-/-*^ mouse model. Deletion of *Ifi35* abated severe weight loss following H5N1- and H1N1-IAV infection. The reduction in weight loss after H5N1-IAV was associated with a decrease in cellular infiltration and production of IL-12p40, CXCL1, and G-CSF. TLR stimulation of *Ifi35*^*-/-*^ mice and primary bone marrow macrophages led to reduced IL-12p40 levels in serum and culture supernatant, respectively; showing that Ifi35 modulates inflammatory responses to multiple stimuli *in vivo* and *in vitro*. In addition, decreased levels of the IL-12p40 homodimer, IL-12p80, was found in bronchoalveolar lavage fluid of H5N1-IAV infected *Ifi35* deficient mice. Depletion of IL-12p80 in WT mice led to reduced cellular infiltration in the lung and protection from severe weight loss following H5N1-IAV infection. Together, our studies suggest that Ifi35 has an early immunomodulatory role that promotes inflammatory responses, such as IL-12p40 and IL-12p80 production, leading to more severe inflammation and disease during H5N1-IAV infection.

## Results

### Ifi35 deficiency leads to reduced weight loss following IAV infection

*Ifi35* expression was previously shown to be associated with increased susceptibility to H5N1-IAV in mice [17]. To study the function of Ifi35 during H5N1-IAV disease progression *in vivo*, we obtained *Ifi35*^*-/-*^ mice that were created by the Knockout Mouse Project Repository (KOMP; Ifi35^tm1(KOMP)Vlcg^). Successful deletion of *Ifi35* was verified by PCR (S1A Fig). Additionally, bone marrow derived macrophages (BMDMs) from C57Bl/6N (WT; wild type) and *Ifi35*^*-/-*^ mice show no evidence of *Ifi35* mRNA expression at baseline or following IFN-β or IFN-γ stimulation (S1B Fig). Phenotypically, the *Ifi35*^*-/-*^ mice are normal and the baseline absolute cell number and the percentage composition of immune cell subsets in naïve WT and *Ifi35*^*-/-*^ lymph nodes, spleen, and thymus are the same for lymphocytes (B cells, CD4+, and CD8+ T cells) or myeloid cell populations (dendritic cells, macrophages, and neutrophils) (S1C–S1E Fig).

**Fig 1:**
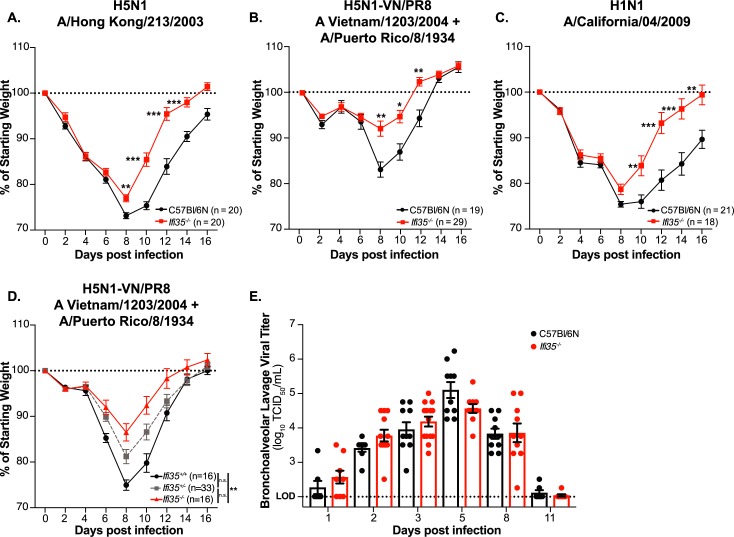
*Ifi35* expression contributes to increased morbidity following avian H5N1 and human H1N1 influenza infection. **(A)** C57Bl/6N (WT; black) or *Ifi35*^-/-^ (red) mice were intranasally infected with 10^4^ TCID_50_ of highly pathogenic H5N1 (A/Hong Kong/213/2003), or **(B)** 100 EID_50_ of H5N1-Vietnam/PR8 (H5N1-VN/PR8; described in Materials and Methods), or **(C)** 10^4^ TCID_50_ of pandemic H1N1 A/California/04/2009 and monitored for weight loss. **(D)** Littermate *Ifi35*^+/+^ (black), *Ifi35*^+/-^ (grey), or *Ifi35*^-/-^ (red) mice were infected with 100 EID_50_ of H5N1-VN/PR8 and monitored for weight loss. Starting weight is indicated with a dashed line at 100%. Values are the means of at least three independent experiments ± SEM. n = 18–29 per group **(E)** Viral titer (log_10_ TCID_50_/mL) in bronchoalveolar lavage fluid from WT (black circle) or *Ifi35*^*-/-*^ (red circle) mice at the indicated time-points post infection. n = 8–14 mice for each time-point from at least three independent experiments. Each symbol represents an individual mouse. Bars represent the mean from at least three different experiments (± SEM). Assay limit of detection (LOD) indicated at 1x10^2^ TCID_50_/mL; dotted line. *P < 0.05, **P < 0.001, ***P < 0.0001 using multiple student t-test for **(A-C)** and **(E)**, One-way ANOVA multiple-comparison for **(D)**.

To assess the impact of Ifi35 deficiency on IAV pathogenesis, we intranasally inoculated WT and *Ifi35*^*-/-*^ mice with 10^4^ TCID_50_ of highly pathogenic avian H5N1-IAV (A/Hong Kong/213/2003) and monitored weight loss and survival (Fig 1A, S2A Fig). *Ifi35*^*-/-*^ mice exhibited decreased weight loss compared to WT animals at 8, 10, and 12 days post infection (dpi) (*P* < 0.001). *Ifi35*^*-/-*^ mice infected with 100 EID_50_ of a 6+2 H5N1 reassortant virus (A/Vietnam/1203/2004 + A/Puerto Rico/8/1934; H5N1-VN/PR8) recapitulate the decreased weight loss phenotype compared to WT mice (*P* < 0.05 for 8 dpi, *P* < 0.01 for 10 and 12 dpi) (Fig 1B). Challenge with the pandemic human H1N1-IAV (A/California/04/2009) also resulted in significantly reduced weight loss in *Ifi35*^*-/-*^ mice (*P* < 0.05 for 8 dpi and *P* < 0.01 for 10 and 12 dpi) (Fig 1C). Finally, *Ifi35*^*+/+*^ *and Ifi35*^*-/-*^ littermates challenged with H5N1-VN/PR8 replicated our WT and *Ifi35*^*-/-*^ weight loss phenotypes (Fig 1D). Additionally, at an institutional cut-off of 30% weight loss, no significant survival differences were observed between WT or *Ifi35*^*-/-*^ mice following either avian or human IAV infection challenge (S2A–S2D Fig). These results show that *Ifi35* expression promotes the progression of disease following both avian and human IAV infection.

### Ifi35^-/-^ and WT mice have similar lung viral load following H5N1-VN/PR8 infection

Decreased weight loss following IAV infection may be due to reduced viral load or altered immune responses in *Ifi35*^*-/-*^ mice. To determine if *Ifi35* expression affected viral titer after infection, we quantified the virus load in bronchoalveolar lavage fluid (BALF) of *Ifi35*^*-/-*^ and WT mice at 1, 2, 3, 5, 8, and 11 dpi with H5N1-VN/PR8. At all time-points tested, the virus titer in BALF was similar between *Ifi35*^*-/-*^ and WT mice (*P* > 0.05) (Fig 1E). In addition to the BALF, homogenized whole lung tissues from the same animals were also titrated and no difference in virus titer was observed between WT and *Ifi35*^*-/-*^ lung homogenates (S2E Fig). Additionally, histopathological assessment of lung consolidation in WT and *Ifi35*^*-/-*^ mice at 5 dpi did not reveal significant differences in total lung area impacted by infection however, *Ifi35*^*-/-*^ mice trended towards less total area of consolidation (S2F–S2G Fig). These data demonstrate that Ifi35 does not overtly affect IAV replication or lung pathology *in vivo* and that the decrease in weight loss in *Ifi35*^*-/-*^ mice after IAV infection is caused by a change in the host response to IAV infection.

### Reduced early immune cell infiltration into lungs of infected Ifi35^-/-^ mice

**Fig 2:**
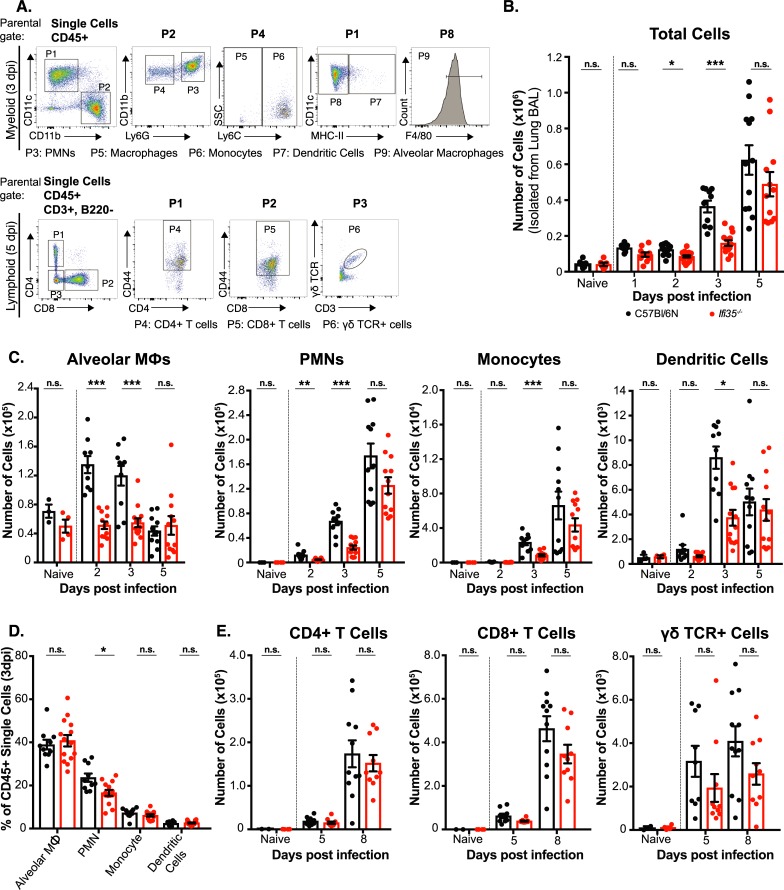
Decreased cellular infiltration into lungs of *Ifi35* deficient mice after H5N1-VN/PR8 infection. **(A)** Gating strategy for analysis of myeloid and lymphoid cells from bronchoalveolar lavage (BAL) of naïve and H5N1-VN/PR8 infected mice. Cells were gated based on CD11b, CD11c, Ly6G, Ly6C, F4/80, and MHC-II expression for myeloid populations or B220, CD3, CD4, CD8, γδ-TCR, and CD44 expression for lymphoid populations. The parental gate is shown above each plot. Representative plots are shown from a WT mouse at 3 dpi for myeloid gating or 5 dpi for lymphoid gating. **(B)** Total number of cells isolated from bronchoalveolar lavage from naïve, infected WT (black), and *Ifi35*^-/-^ (red) mice at indicated times post infection. **(C)** Total number of CD45^+^ alveolar macrophage (CD11c^+^, CD11b^-^, MHC-II^-^, F4/80^+^), neutrophil (PMNs; CD11b^+^, CD11c^-^, Ly6G^+^), monocyte (CD11b^+^, Ly6G^-^, Ly6C^hi^), and dendritic cell (CD11c^+^, CD11b^-^, MHC-II^+^) numbers in BAL. **(D)** Percentage of myeloid cell populations in BAL at 3 dpi following pre-gating on CD45^+^ cells. **(E)** Total number of CD3^+^, CD44^+^ lymphoid CD4^+^, CD8^+^, or γδ-TCR^+^ T cells in BAL. n = 8–11 for each group/time point post-infection. n = 3–4 for naïve samples. Each symbol represents an individual mouse. Bars represent the mean from at least three different experiments (± SEM). PMN, polymorphonuclear cells; n.s., not significant; *P < 0.01, **P < 0.001, ***P < 0.0001 using multiple student t-test.

Next, we characterized the kinetics of immune cell infiltration into the lungs of WT and *Ifi35*^*-/-*^ mice following H5N1-VN/PR8 infection. Cells were separated from BALF, counted, and surface marker-stained for identification of cell subsets by flow cytometry (Fig 2A). At baseline, similar numbers of total viable cells were isolated from naïve *Ifi35*^*-/-*^ and WT lungs. Following infection, however, the BAL of *Ifi35*^*-/-*^ mice contained fewer cells at 2 and 3 dpi (*P* < 0.01), but not at 1, 5, 8, or 11 dpi (*P* > 0.1) (Fig 2B and S3A Fig). Next, we sought to determine if a specific subset of cells contributed to this phenotype. The numbers of alveolar macrophages (CD11c^+^, F4/80^+^, MHC-II^-^) and neutrophils (CD11b^+^, Ly6G^+^) were significantly reduced at 2 and 3 dpi, while dendritic cells (CD11c^+^, MHC-II^+^, Ly6G^-^, F4/80^-^) and inflammatory monocytes (CD11b^+^, Ly6C^hi^, Ly6G^-^) were only reduced at 3 dpi (Fig 2C and S3B Fig). Furthermore, the overall percentage of neutrophils (CD11b^+^, Ly6G^+^) in *Ifi35*^*-/-*^ mice was significantly (*P <* 0.01) reduced at 3 dpi (Fig 2D). We also evaluated lymphoid cell subsets by characterizing CD4^+^, CD8^+^, and γδ-TCR^+^ T cells in the lungs after infection. The numbers of all infiltrating T cell subsets were significantly lower in *Ifi35*^*-/-*^ mice at 3 dpi, but not any other time point including 8 and 11 dpi when adaptive immunity is critical for IAV control (Fig 2E and S3C Fig). These results show that Ifi35 potentiates early recruitment of pro-inflammatory immune cells to the lung following IAV infection.

### Reduced early production of cytokines and chemokines following H5N1-VN/PR8 infection in Ifi35^-/-^ mice

**Fig 3:**
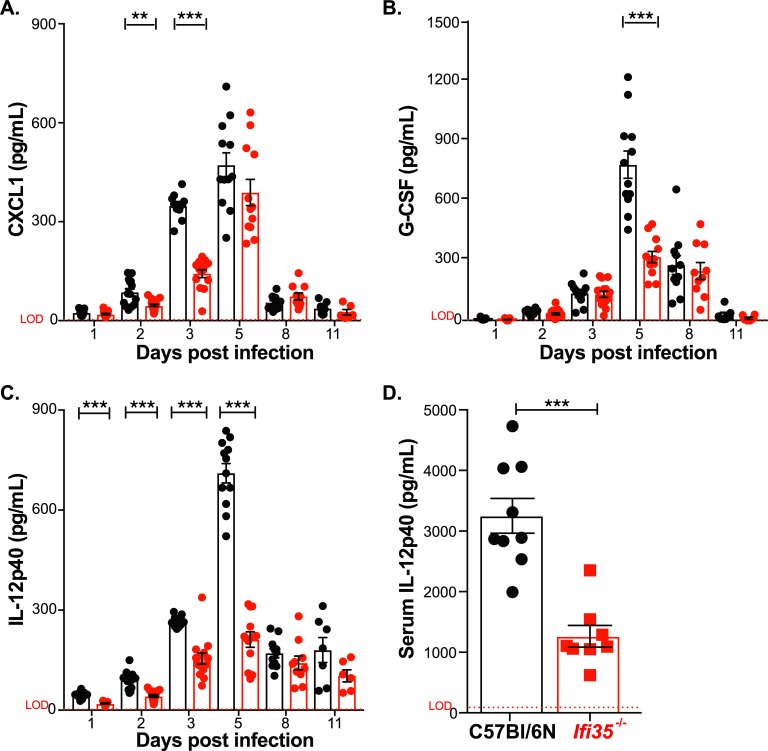
Specific cytokines and chemokines are significantly reduced in *Ifi35*^-/-^ mice at early time-points following H5N1-VN/PR8 infection and *in vivo* poly I:C challenge. **(A)** CXCL1, **(B)** G-CSF, and **(C)** IL-12p40 concentrations (pg/mL) in bronchoalveolar lavage fluid from WT (black circle) and *Ifi35*^-/-^ mice (red circle) at indicated times post infection with H5N1-VN/PR8 as measured by Bio-Plex Pro Mouse Cytokine 23-Plex assay. Dotted line (red) indicates limit of detection (LOD) for individual cytokines and chemokines based on internal standard curve values. **(D)** Serum IL-12p40 concentration (pg/mL) in WT (black circle) and *Ifi35*^*-/-*^ (red square) mice at 3 hours post stimulation with 100μg HMW poly I:C via intraperitoneal injection. Serum cytokine levels measured using an IL-12p40 specific ELISA; dotted line indicates LOD based on standard curve values. Each symbol represents an individual mouse. Bars represent the mean from at least three different experiments (± SEM). n = 6–19 per group per time-point. *P < 0.01, **P < 0.001, ***P < 0.0001 using multiple student t-test.

The reduction in immune cell infiltration into the lungs of *Ifi35*^*-/-*^ mice could be due to altered cytokine and chemokine responses following H5N1-VN/PR8 infection. Analysis showed that only three cytokines and chemokines were significantly different in BALF from *Ifi35*^*-/-*^ and WT mice (Fig 3, S1 Table). The neutrophil chemoattractant protein, CXCL1 (KC), was significantly reduced in *Ifi35*^*-/-*^ mice at 2 and 3 dpi (*P* < 0.001; Fig 3A) and G-CSF was reduced at 5 dpi in *Ifi35*^*-/-*^ mice (*P* < 0.0001; Fig 3B). Importantly, *Ifi35*^*-/-*^ mice had significantly reduced levels of IL-12p40 as early as 1 dpi (*P* < 0.001; Fig 3C) compared to WT mice and this difference was sustained until 5 dpi (*P* < 0.0001). Of note, the amount of IL-12p70 (IL-12) was similar between WT and *Ifi35*^*-/-*^ mice, suggesting that the reduced IL-12p40 levels in *Ifi35*^*-/-*^ mice do not affect IL-12p70 levels, an important cytokine for Th1 immune responses (S4A Fig). In agreement with this data, we did not observe a difference in IFN-γ production or infiltration of CD4+ and CD8+ T-cells on 8 and 11 dpi between WT and *Ifi35*^*-/-*^ mice (S1 Table, Fig 2E and S3C Fig). Production of IL-17, a cytokine downstream of IL-23 (IL-12p40/p19 heterodimer) and important for Th17 immunity, was also not significantly different between WT and *Ifi35*^*-/-*^ mice (S4B Fig and S1 Table).

Given that we observed a sustained reduction of IL-12p40 production in *Ifi35*^*-/-*^ mice starting on 1 dpi, we sought to ask if the effect of Ifi35 on IL-12p40 production *in vivo* was specific for H5N1-VN/PR8 infection. WT and *Ifi35*^*-/-*^ mice were challenged with 100μg of high molecular weight poly I:C (HMW poly I:C) intraperitoneally for 3 hours before serum collection and cytokine analysis. *Ifi35*^*-/-*^ mice had lower serum levels of IL-12p40 compared to WT mice (*P* < 0.0001) (Fig 3D), suggesting that Ifi35 augments IL-12p40 production in response to inflammatory stimuli.

Ifi35 has previously been reported to alter type I IFN production *in vitro* [19–21]. To investigate the effects of Ifi35 on type I IFN expression *in vivo*, we quantified the amount of type I IFN in BALF from WT and *Ifi35*^*-/-*^ mice after H5N1-VN/PR8 infection using a highly sensitive L929 and EMCV cell bioassay [29]. *Ifi35*^*-/-*^ and WT mice had similar levels of type I IFN in their lungs at 2, 3, and 5 dpi (*P* > 0.15), showing that, in the context of IAV infection, Ifi35 does not affect type I IFN production (S4C Fig).

### Hematopoietic cell expression of Ifi35 increases cellular infiltration and IL-12p40 levels in the lung following H5N1-VN/PR8 infection

**Fig 4:**
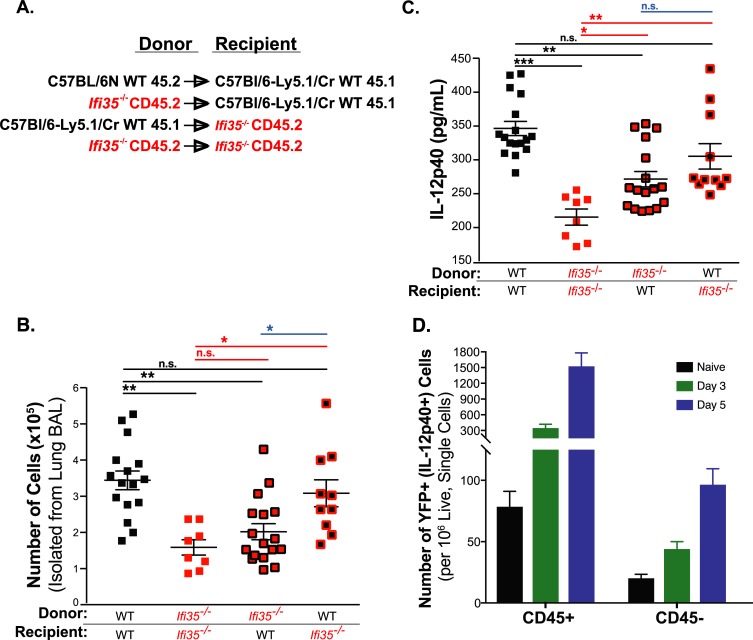
*Ifi35* expression in hematopoietic cells promotes cellular recruitment into the lung and IL-12p40 production following H5N1-VN/PR8 infection. **(A)** CD45.1^+^ B6-Ly5.1/Cr and CD45.2^+^ *Ifi35*^-/-^ mice were Busulfan treated and reconstituted with CD45.1^+^ B6-Ly5.1/Cr, CD45.2^+^ WT, or CD45.2^+^ *Ifi35*^-/-^ bone marrow cells (10^7^ cells). Bone marrow donor and recipient pairs for the experiment are shown. **(B)** Twelve-week-old chimeric mice were infected with 100 EID_50_ of H5N1-VN/PR8 and BAL fluid was collected at 3 dpi and assessed for total BAL cell number or **(C)** assessed for IL-12p40 levels via ELISA. Each symbol represents an individual mouse. Bars represent the mean from at least three different experiments (± SEM). n = 8–17 per group. **(D)** IL-12p40-IRES-eYFP reporter (IL12b-yet40) mice were infected with 100 EID_50_ of H5N1-VN/PR8 and on 0, 3, and 5 dpi whole lungs were taken for collagenase digestion and flow cytometry analysis of eYFP expression (FITC^+^) and CD45 surface staining. FITC^+^ cells were separated based on CD45 staining and enumerated per 1x10^6^ single cells collected. Bars represent the mean from at least three different experiments (± SEM). n = 4 for naïve and 11–12 for infected groups. n.s., not significant; *P < 0.01, **P < 0.001, ***P < 0.0001, using one-way ANOVA with multiple comparisons correction (Tukey’s comparison correction).

We next assessed whether Ifi35 expression in hematopoietic cells was necessary for cellular recruitment to the lung and IL-12p40 induction following infection. To address this, we generated reciprocal bone marrow (BM) chimeras using CD45.1^+^ and CD45.2^+^ donor and recipient mice and measured cellular infiltration and IL-12p40 levels at 3dpi with H5N1-VN/PR8 (Fig 4A). The total number of cells in the BALF of WT mice that had received *Ifi35*^*-/-*^ BM (*Ifi35*^*-/-*^ → WT) was significantly lower compared to WT control transfer (WT → WT) (*P <* 0.001) (Fig 4B). In contrast, *Ifi35*^*-/-*^ recipient mice that had received BM from WT mice (WT → *Ifi35*^*-/-*^) had significantly more cells in the BALF compared to the *Ifi35*^*-/-*^ control transfer (*Ifi35*^*-/-*^ → *Ifi35*^*-/-*^) (*P <* 0.05), suggesting that cellular infiltration at 3 dpi is dependent on Ifi35 expression in the hematopoietic cell compartment (Fig 4B). Similarly, IL-12p40 production in the mixed BM animals (WT → *Ifi35*^*-/-*^ and *Ifi35*^*-/-*^ → WT) was significantly higher (*P <* 0.001) and lower (*P <* 0.001), respectively, compared to the control transfers (Fig 4C). However, the amount of IL-12p40 produced in the mixed BM mice was intermediate to that of the homologous BM transfer mice; showing that the production of IL-12p40 at 3 dpi depends on Ifi35 expression in both hematopoietic and non-hematopoietic cells.

The expression of IL-12p40 by hematopoietic and non-hematopoietic cells after IAV infection was confirmed using the IL-12b-eYFP (yet40) reporter mouse [30]. After H5N1-VN/PR8 infection, the majority of the IL-12p40 (i.e. YFP^+^) expressing cells were of hematopoietic cell origin (CD45^+^). However, a significant number of non-hematopoietic cells (CD45^-^) also expressed IL-12p40 after infection and this number increased between 3 and 5 dpi (Fig 4D and S5 Fig).

### Ifi35 deficiency reduces IL-12p40 concentration following TLR stimulation

**Fig 5:**
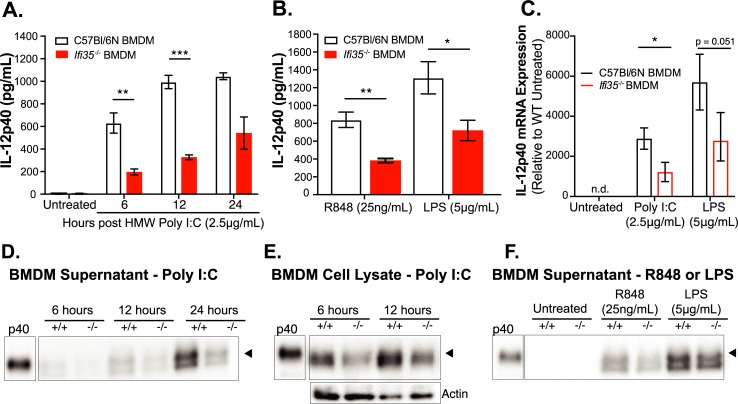
*Ifi35* expression in bone marrow derived macrophages increases IL-12p40 supernatant levels following TLR stimulation. **(A)** Supernatant IL-12p40 levels at different time points post treatment from bone marrow derived macrophages (BMDMs) stimulated with 2.5**μ**g/mL HMW poly I.C. **(B)** Supernatant IL-12p40 levels following either R848 (25ng/mL) or LPS (5**μ**g/mL) treatment for 12 hrs. n = 3–6 per group from at least three independent experiments. **(C)** BMDM IL-12p40 mRNA levels measured by qRT-PCR following poly I:C or LPS treatment for 6 hrs. Values are fold change relative to C57Bl/6N untreated samples. n = 3 per group from three independent experiments. **(D)** Non-reducing western blot of 10X concentrated BMDM supernatant following HMW poly I:C stimulation at indicated time-points. **(E)** Non-reducing western blot of cell lysate from Brefeldin-A treated BMDMs stimulated with HMW poly I:C. Blot probed for accumulated cellular IL-12p40 and subsequently, β-actin for loading control. **(F)** Non-reducing western blot of 10X concentrated BMDM supernatant 12hrs following either LPS or R848 treatment. Supernatant IL-12p40 levels were measured using an IL-12p40 specific ELISA. Western blots were probed with anti-IL-12p40 antibody and run with rIL-12p40 as a standard. All graphed values are the means ± SEM from at least three independent experiments. Original, uncropped blot images are shown in S6 Fig. *P < 0.01, **P < 0.001, ***P < 0.0001, using multiple student t-test.

To investigate how Ifi35 expression modulates IL-12p40 production following TLR signaling in immune cells, we stimulated BMDMs from WT and *Ifi35*^*-/-*^ mice with TLR-3, TLR-4, or TLR-7 agonists and measured IL-12p40 production. When WT or *Ifi35*^*-/-*^ BMDMs were stimulated with 2.5μg/mL of HMW poly I:C, we observed decreased levels of IL-12p40 in cell supernatants from *Ifi35*^*-/-*^ BMDMs at 6 and 12 hours post-stimulation (hpi) using an IL-12p40 specific ELISA (6hpi, *P* < 0.01; 12hpi, *P* < 0.0001) (Fig 5A). Stimulation with Resiquimod (R848) or lipopolysaccharide (LPS), also led to significantly less IL-12p40 production by *Ifi35*^*-/-*^ BMDMs (R848, *P* < 0.01; LPS, *P* < 0.05) (Fig 5B). Quantitative RT-PCR analysis of *IL-12p40* mRNA from BMDMs following poly I:C stimulation revealed a 2.5-fold decrease (*P* < 0.05) in *Ifi35*^*-/-*^ BMDMs compared to WT BMDMs (Fig 5C). The differences in IL-12p40 protein production were also confirmed by western blot on concentrated supernatant samples or whole cell lysates (Fig 5D–5F). These data show that in the absence of Ifi35 expression, IL-12p40 concentrations in primary BMDM supernatant is significantly reduced after TLR stimulation.

### IL-12p80 is a major IL-12p40 species present in BALF following H5N1-VN/PR8 infection

**Fig 6:**
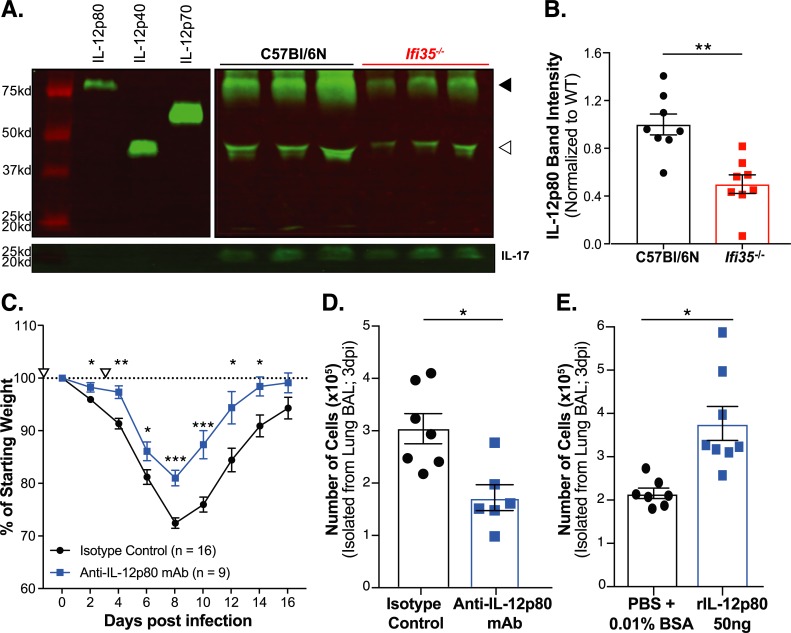
Therapeutic IL-12p80 blockade leads to reduced cellular recruitment and weight loss upon H5N1-VN/PR8 infection. **(A)** Representative image of IL-12p40 species (anti-IL12p40 mAb) in WT and *Ifi35*^-/-^ bronchoalveolar lavage fluid (BALF) at 5 dpi visualized by western blotting under non-reducing conditions and detected by fluorescent secondary antibody (Alexa Fluor 790 goat anti-rat). Bands that correspond to the IL-12p40 homodimer (IL-12p80) and IL-12p40 monomer (IL-12p40) are indicated by solid and open arrowheads, respectively. IL-17 was used as loading control given similar levels of production in both WT and *Ifi35*^*-/-*^ mice in two independent assays (S4 Fig and S1 Table). **(B)** Mean band intensity of IL-12p80 and IL-12p40 bands quantified relative to IL-17 production using LiCor Image Studio software for WT (n = 8) and *Ifi35*^-/-^ mice (n = 8). **(C)** Weight loss in H5N1-VN/PR8 infected WT mice that received 100μg of either anti-IL-12p80 mAb a3-1d (blue line) or Armenian Hamster IgG (black line) on day -1 and day +3 (indicated by open triangle). n = 9–16 per group. **(D)** Total number of cells in BAL on day 3 post infection from H5N1-VN/PR8 infected WT female mice that received either 100μg anti-IL-12p80 mAb a3-1d (blue squares) or 100μg Armenian Hamster IgG (black circles) at day -1. n = 6–7 per group. **(E)** Total number of cells in BAL on day 3 post infection from H5N1-VN/PR8 infected WT male mice that received either 50μL PBS/0.01% BSA (black circles) or 50ng recombinant IL-12p80 in 50μL (blue squares) intranasally at 1 dpi. n = 7–8 per group. Each symbol represents an individual mouse in D-E. All graphed values are the means ± SEM from at least three **(B, C)** or two **(D, E)** independent experiments. Original, uncropped blot images are shown in S6 Fig. *P < 0.05, **P < 0.001, ***P < 0.0001 using multiple student t-test.

IL-12p40 can function as a monomer (p40), homodimer (p80), or heterodimer (IL-12p70 and IL-23) [31–33]. Each cytokine has a unique signaling pathway and effect on inflammation and disease progression. Since we observed no difference in IL-12p70 and IL-17 production, which is dependent on IL-23, between the WT and *Ifi35*^*-/-*^ mice, we quantified IL-12p40 and IL-12p80 production in BALF from WT and *Ifi35*^*-/-*^ mice at 5 dpi after H5N1-VN/PR8 by western blot (Fig 6A). These analyses showed that approximately 60% of total IL-12p40 is present as the IL-12p80 homodimer (S6D–S6E Fig). Furthermore, the amounts of IL-12p40 and IL-12p80 were significantly reduced in BALF from H5N1-VN/PR8 infected *Ifi35*^*-/-*^ mice compared to BALF from WT animals (*P* < 0.01) (Fig 6B). This is the first description of IL-12p80 in BALF following IAV infection and suggests a model wherein Ifi35 modulates IL-12p40 and IL-12p80 production in the context of IAV infection.

### Functional blockade of IL-12p80 ameliorates weight loss and reduces cell recruitment into the lung following H5N1-VN/PR8 infection

To determine if IL-12p80 production exacerbates disease after IAV infection, WT mice were treated with a specific anti-IL-12p80 antibody or isotype control antibody at day -1 and day +3 following H5N1-VN/PR8 infection. Mice receiving the anti-IL-12p80 specific antibody had significantly reduced weight loss at early time-points following infection (2 dpi and 4 dpi; *P* < 0.05) and reduced peak weight loss at 8 dpi (*P* < 0.01) compared to the animals receiving the isotype control (Fig 6C). There were no survival difference between mice receiving the anti-IL-12p80 specific antibody and mice receiving isotype control (S6F Fig). Treatment of WT mice with anti-IL-12p80 at day -1 led to significantly fewer cells in BALF at 3 dpi; resembling what we initially observed in *Ifi35*^*-/-*^ mice (Fig 6D). Furthermore, intranasal treatment of WT mice with exogenous recombinant IL-12p80 on 1 dpi led to significantly increased cells in BALF at 3 dpi; showing that IL-12p80 promotes cellular recruitment to the lung (Fig 6E). These results demonstrate that the reduced cellular recruitment and weight loss in *Ifi35*^*-/-*^ mice is, in part, due to reduced levels of IL-12p80 following H5N1-VN/PR8 infection.

## Discussion

Infection with H5N1-IAV induces a robust inflammation characterized by an amplified cytokine response leading to immunopathology and severe disease. The expression of *Ifi35* during H5N1-IAV infection in mice enhanced weight loss, early cellular infiltration into the lung, and production of IL-12p40, IL-12p80, CXCL1, and G-CSF. The increased IL-12p80 concentration in WT mice was deleterious, as treatment with anti-IL-12p80 antibody reduced weight loss following H5N1-IAV infection.

The significance of IL-12p40 and IL-12p70 during IAV infection and pathogenesis has been extensively studied using different gene knockout mice [34,35] or neutralizing antibodies specific for IL-12p40 or IL-12p35 [36–39]. However, the outcomes from these studies are ambiguous and suggest both protective and deleterious effects of IL-12 (IL-12p70) on IAV disease. Interrogating the role of IL-12p40 is also complicated by its multiple binding partners. IL-12p40 monomer associates with IL-12p35 to generate functional IL-12p70, with IL-12p19 to form IL-23, and homodimerizes to form IL-12p80.

IL-12p40 is required for the production of heterodimeric IL-12p70 and IL-23. Despite an observed reduction in IL-12p40, IL-12p70 and IL-23 were the same between WT and *Ifi35*^*-/-*^ mice (as measured directly or through IL-17, a downstream effector cytokine of IL-23, respectively). Since IL-12p40 is produced in vast excess of IL-12p35 and IL-12p19, the production of IL-12p70 and IL-23 is dependent on the respective partner subunit [40,41]. Therefore, a 2–3 fold reduction in IL-12p40 protein does not affect IL-12p70. The rationale for the surplus of IL-12p40 is not well understood, but perhaps the formation of IL-12p80 and initiation of an immune response may be the reason for this.

In addition to IL-12p35 and IL-12p19, IL-12p40 has been shown bind to several other partners to potentially generate unique cytokines or signaling molecules [42]. Therefore, targeting IL-12p40 monomer may affect multiple facets of the innate immune response. IL-12p40 monomer/homodimer was previously shown to induce Fas-ligand expression on plasmacytoid DCs leading to the apoptosis of IAV-specific CD8+ T-cells through a Fas-FasL mediated pathway [12]. We did not observe any ablation of total CD4^+^, CD8^+^ or γδ-TCR^+^ T-cell numbers in our WT mice following IAV infection despite increased levels of IL-12p40 production at early time-points. Also, we did not observe a difference in viral titer in the lungs of the *Ifi35*^*-/-*^ mice at 8 or 11 dpi. This disparity may be due to differences in challenge dose, mouse strain, or virus strain.

In our study, WT mice infected with H5N1-VN/PR8 had increased amounts of IL-12p40 and IL-12p80 in BALF following H5N1-VN/PR8 infection compared to *Ifi35*^*-/-*^ mice, suggesting that IL-12p80 may contribute to more severe weight loss; a characteristic of IAV disease. In mice, IL-12p80 production is associated with severe Sendai virus infection and worsened experimental autoimmune encephalomyelitis (EAE) [43,44]. More recent studies have shown that IL-12p80 is a potent chemo-attractant for macrophages and dendritic cells and can induce nitric oxide production in macrophages and microglia [45–48]. Nitric oxide has been shown to contribute to more pathogenic outcomes of H5N1 and 1918 H1N1 IAV infection [49]. Significantly, the neutralization of IL-12p80 *in vivo* during H5N1-VN/PR8 infection (Fig 6C) or EAE, ameliorates severe disease [44]. These data support a critical role for IL-12p80 in exacerbating inflammatory diseases, including H5N1-IAV. Therefore, by using a specific IL-12p80 targeting antibody, we directly tested whether IL-12p80 is pathogenic during IAV infection and showed that this homodimer has pathogenic potential and contributes to increased cellular infiltration following IAV infection. These findings provide novel insight into the role of IL-12p40 and IL-12p80 during IAV pathogenesis.

*Ifi35*^*-/-*^ mice produced less IL-12p40 after *in vivo* poly I:C stimulation. Moreover, BMDMs from *Ifi35*^-/-^ mice failed to induce WT levels of IL-12p40 following *in vitro* TLR-3, TLR-4, and TLR-7 stimulation and showed decreased IL-12p40 mRNA levels following TLR-3 and TLR-4 stimulation. This suggests that Ifi35 acts on a shared feature of the TLR signaling pathway to increase IL-12p40 gene expression. Currently it is not known how Ifi35 modulates IL-12p40 expression. The Ifi35 protein contains an atypical helix-loop-helix (leucine zipper) domain that lacks the basic DNA binding region and thus is unlikely to bind directly to DNA and enhance IL-12p40 expression. The nuclear localization of Ifi35 upon activation [23,24] and its reported interactions with CKIP-1, a regulator of AP-1 family transcription factors and B-ATF, an AP-1 family transcription factor, suggest roles in the transcriptional regulation of cytokine production and specifically, of IL-12p40 [23,27,50]. The second characterized domain of Ifi35, the NMI/IFI35 homology domain (NID), has been shown to facilitate the interaction between Ifi35 and NMI (N-myc and STAT interactor protein) [22,25,26,51]. Since NMI is known to interact with Stat1 and Stat5, the interaction of Ifi35 with NMI may alter downstream signaling through these transcription factors [52].

Ifi35 has been shown to induce pro-inflammatory cytokines and immune responses in multiple models. Recently, extracellular Nmi and Ifi35 were reported to act as a DAMP (damage-associated molecular pattern) and activate macrophages to release pro-inflammatory cytokines through NF-κB activation and TLR-4 pathway signaling [28]. Ifi35 has also been shown to positively regulate TLR-3 dependent CCL5 and CXCL10 expression and exacerbate the pathogenesis of glomerular inflammation [53]. Additionally, Ifi35 overexpression affected endothelial cell proliferation, migration, and re-endothelialization after injury through the inhibition of the NF-κB/p65 pathway, suggesting that the context of Ifi35 expression may determine its function in regulating inflammatory responses [54].

*Ifi35* mRNA expression in the lung following H5N1 infection showed a significant induction at 3 dpi (*P* < 0.0039) when compared to uninfected mice. However, since we see reduced IL-12p40 as early as 1 and 2 dpi, it is likely that basally expressed Ifi35 has an impact on initial immune responses. Publicly available mRNA expression data from mouse and human samples suggest that Ifi35 is expressed at baseline in lung tissues [55,56]. Additionally, the Knock-Out Mouse Project, through which the *Ifi35*^*-/-*^ mouse was made, characterized protein expression in various organs through the use of a LacZ reporter and show expression in naïve lung tissue. Finally, our *in vivo* poly I:C stimulation shows reduced IL-12p40 serum levels at 3 hours post-infection and provides evidence for a role of basally expressed Ifi35 protein.

Murine Ifi35 and human Ifi35 are 72% identical at the amino acid level. The basic leucine zipper domain and the NID domains are conserved between both murine and human Ifi35, therefore it is possible that they have a similar function during inflammation. In human non-viral inflammatory diseases, increased levels of IL-12p80 have been reported as being pathogenic. BALF from asthmatic human subjects, showed increased levels of IL-12p80 compared to BALF from normal patients and this was associated with increased numbers of BALF macrophages. Additionally, human lung transplant recipients with transplant bronchitis showed excessive IL-12p80/IL-12p40 production in BALF and increased macrophage accumulation that the authors attributed to the chemo-attractant properties of IL-12p80 [57,58]. IL-12p40 homodimer was also reported to be present in high concentrations (ng/mL) in serum from healthy control patients while reduced or undetectable in the serum of prostate cancer patients [59]. These studies suggest an important role for IL-12p80 in non-viral inflammatory diseases. Our study is the first to provide a role for murine Ifi35 in modulating inflammatory response and IL-12p40/IL-12p80 production following virus infection in mice. Whether IL-12p80 is produced during influenza virus infection in humans will be addressed in future studies.

Ifi35 has also been suggested to increase or decrease type I IFN in response to virus infection in *in vitro* culture settings [19–21]. Importantly, Ifi35 over-expression has been reported to inhibit RIG-I function and thus, reduce IFN-β production following vesicular stomatitis virus (VSV) and Sendai virus infection [21]. Our study is the first to interrogate the function of Ifi35 during virus infection *in vivo*. We did not observe an effect of Ifi35 expression on type I IFN production in our model. One possible explanation for this difference is that IAV viral proteins antagonize the RIG-I pathway [60,61] and thus, any additional inhibitory effects of Ifi35 on this pathway will not be observed in this model. Alternatively, the function of Ifi35 is different or more complex in an *in vivo* setting during viral infection. Finally, it is possible that the effects of Ifi35 on type I IFN expression occur at an earlier time point after infection than what we interrogated. Additional experiments detailing the early type I IFN kinetics and analyzing the transcriptional profile of whole lung tissues following IAV infection may elucidate a role of Ifi35 in IFN regulation *in vivo*.

In summary, our study reveals a profound role of Ifi35 in modulating IL-12p40 and IL-12p80 cytokine production during H5N1-VN/PR8. We show that Ifi35 expression in hematopoietic cells facilitates increased cellular infiltration largely through IL-12p40 induction in the lung, ultimately leading to severe weight loss. The increased presence of IL-12p80, the IL-12p40 homodimer, is sufficient for increased weight loss in WT mice following H5N1-IAV infection as specific inhibition of IL-12p80 ameliorated severe weight loss in WT mice. These studies have brought to light the significant pathogenic contribution of IL-12p40 and IL-12p80 in H5N1-IAV disease progression, an observation that may provide insight into the development of therapeutic agents to ablate the inflammatory pathology that leads to severe IAV disease.

## Methods

### Ethics statement

Animal studies were carried out in strict accordance with the recommendations in the Guide for the Care and Use of Laboratory Animals of the National Institutes of Health. The Institutional Animal Care and Use Committee (IACUC) at the Washington University School of Medicine approved all animal protocols (protocol number: 20160261). Intranasal inoculation and retro-orbital injections were performed under anesthesia with Avertin (2,2,2-tribromoethanol; Sigma-Aldrich, MO; 250mg/kg dosage) or Isoflurane inhalation, respectively, and all efforts were made to minimize animal discomfort.

### Mice

C57Bl/6N (WT) mice were bred in-house in a barrier facility and received food and water *ad libitum* in accordance with IACUC protocols. IL12b-yet40 homozygous mice (stock #006412; p40-IRES-eYFP) were purchased from Jackson Laboratories and bred in-house. Adult female mice (6- to 8-week-old) were used for survival, weight loss, cellular infiltration, cytokine, and anti-IL-12p80 antibody administration weight loss experiments. BAL experiments following anti-IL-12p80 or rIL-12p80 administration were performed with female and male mice. BMDM cultures were derived from adult male mice (6 to 8-week-old) and *in vivo* poly I:C stimulations were performed on adult male mice (6 to 8-week-old).

### Generation and genotyping of *Ifi35*^*-/-*^ mice

*Ifi35*^*-/-*^ mice (Ifi35^tm1(KOMP)Vlcg^; C57Bl/6N background) generated by Velocigene technology through the Knockout Mouse Project Repository (KOMP, University of California, Davis) were purchased and subsequently bred in-house. In brief, the *Ifi35* gene (Ifi35; NCBI gene ID: 70110) was replaced by a reporter-selection cassette, which consists of LacZ and a neomycin resistance gene. Mice were genotyped by PCR using the following primer pairs provided by KOMP: TDF: GGGCCTGGCTATCTTCACTTC, TDR: TGGCACTTTGACCGTTCTTG, LacZInf: GGTAAACTGGCTCGGATTAGGG, LacZInR: TTGACTGTAGCGGCTGATGTTG where *Ifi35*^*-/-*^ mice produce a PCR product of 210 bp, *Ifi35*^+/+^ mice produce a PCR product of 105bp, and *Ifi35*^+/-^ mice produce a PCR product of both 210 bp and 105 bp. These primers were also used in-house for genotyping *Ifi35*^+/-^ x *Ifi35*^+/-^ offspring for littermate control experiments.

### Cellular characterization of naïve *Ifi35*^*-/-*^ mice

Lymph nodes (axillary, brachial, and inguinal), thymus, and spleen were harvested from 6- to 8-week-old naïve male and female C57Bl/6N and *Ifi35*^*-/-*^ mice. Red blood cell lysed, single-cell suspensions from each tissue type were counted and 1 - 2x10^7^ cells were stained for cellular analysis. Cells were stained in two mixes that included CD3ε (Biolegend, clone 145-2C11), CD45R (BD Pharmingen, clone RA3-6B2), CD8α (BD Pharmingen, catalog 552877), NK1.1 (BioLegend, clone PK136), CD11c (eBioscience, clone N418), CD19 (Biolegend, clone 6D5), CD4 (Biolegend, RM4-5), I-A/I-E (Biolegend, catalog 107626), F4/80 (Biolegend, clone BM8), Ly6C (Biolegend, clone HK1.4), Ly6G (Biolegend, clone 1A8), CD64 (Biolegend, clone X54-5/7.1), CD11b (Biolegend, clone M1/70), and MerTK (R&D Systems, catalog BAF591) which was followed by streptavidin Qdot-605 secondary staining (Invitrogen, catalog Q10101MP). Stained samples were run on a LSRFortessa flow cytometer (BD Biosciences). The resulting data was analyzed using FlowJo software (Treestar).

### Viruses

A highly pathogenic H5N1 (H5N1) influenza A virus was rescued by reverse genetics as described previously [62]. The virus contains seven gene segments from A/Hong Kong/213/2003 H5N1 virus and the PB1 gene segment from A/chicken/Hong Kong/Y0562/2002 H5N1 virus [17,63]. The 6+2 H5N1 reassortant virus (H5N1-VN/PR8) contains the HA and NA genes from the H5N1 IAV strain A/Vietnam/1203/2004 while the other six gene-segments are obtained from A/Puerto Rico/8/1934 virus. The H5 HA from A/Vietnam/1203/2004 is modified to remove the polybasic cleavage site. Human pH1N1 A/California/04/2009 influenza viral stocks were prepared as previously described [64].

### Mouse infections

Six to 8-week-old female C57Bl/6N and *Ifi35*^*-/-*^ mice were intranasally inoculated with either 10^4^ TCID_50_ of highly pathogenic avian H5N1 A/Hong Kong/213/2003 (H5N1), 100 EID_50_ (egg infectious dose 50) of a 6+2 H5N1 reassortant virus (A/Vietnam/1203/2004 + A/Puerto Rico/8/1934; (H5N1-VN/PR8)), or 10^4^ TCID_50_ of pandemic human H1N1 A/California/04/2009 (pH1N1) in 30ul of sterile phosphate-buffered saline (PBS) after sedation with Avertin. Infections with H5N1 were carried out in an approved ABSL3+ facility, according to protocols established and approved by the Centers of Disease Control. Morbidity and mortality was monitored for 20 days and mice were euthanized when percent body weight loss exceeded 30%. For BAL analysis or tissue procurement, mice were infected as described and at 1, 2, 3, 5, 8, or 11 days post-infection were sacrificed by Avertin overdose followed by cervical dislocation.

### *In vivo* HMW poly I:C stimulation

Six to 8-week-old male C57Bl/6N and *Ifi35*^*-/-*^ mice were stimulated with 100μg of high molecular weight poly I:C (HMW poly I:C; Invivogen #tlr3-pic) resuspended in endotoxin-free physiological water (0.9% NaCl; provided by Invivogen) via intraperitoneal injection. Three hours following stimulation, mice were exsanguinated by cardiac puncture under Avertin anesthesia. Blood was allowed to clot at room temperature for 20 minutes and serum was separated by centrifugation at 1000xg for 10 minutes. Aliquots of serum were frozen at -80°C and used for IL-12p40 ELISA.

### Generation of bone marrow chimeric mice

Four to 5-week-old female recipient CD45.1^+^ C57Bl/6-Ly5.1 (Charles River Laboratories) or CD45.2^+^ *Ifi35*^*-/-*^ mice were treated with 125mg/kg of Busulfan (25mg/kg/day for 5 days ending on day -1 prior to bone marrow transfer [65] and 10^7^ donor bone marrow cells from 4- to 5-week-old female CD45.2^+^ (C57Bl/6N or *Ifi35*^*-/-*^) or CD45.1^+^ (C57Bl/6-Ly5.1) donor mice were transferred into myeloablated recipients by retro-orbital injection. Mice were cheek-bled for chimerism analysis by flow cytometry at 8 weeks post bone marrow transfer, prior to infection with 100 EID_50_ of H5N1-VN/PR8. Mice with ≥ 90% donor-derived myeloid cells (neutrophils, macrophages, and dendritic cells) were used for infection experiments. The frequency of donor-derived T- and B-cells in these same mice were 70–75% and 80–85% respectively.

### Bronchoalveolar lavage of mice

For analysis of lung airway cellularity and cytokine and chemokine profiles following infection, mice were sacrificed by Avertin overdose followed by anterior neck dissection and cannulation of the trachea with a 22-gauge angiocatheter. Bronchoalveolar lavage (BAL) was performed with three washes of 0.8 mL of sterile HBSS (Hank’s Balanced Salt Solution; 2.4 mL total BAL fluid/mouse). BAL fluid was centrifuged and the cell-free supernatant was collected, aliquoted, and stored at -80°C until further use for viral titer, cytokine/chemokine, and western blot analysis. The BAL cell pellet was lysed of red blood cells with 0.84% NH_4_Cl pH 7.4, quenched and washed with PBS/2% FBS, and resuspended in 0.5mL PBS/2% FBS for total viable cell count and immuno-phenotyping.

### Flow cytometry

Cells collected from BAL were stained with mAbs specific for CD45, CD3, CD8, CD4, CD44, CD11b, CD11c, Ly6G, Ly6C, MHC-II, γδ-TCR, F4/80, and B220 to define myeloid and lymphoid populations. Refer to Key Resources Table (S2 Table) for full panel of antibodies used in immune cell characterization. Gating strategies are depicted in Fig 2A.

For analysis of cells that produce IL-12p40, whole lungs from H5N1-VN/PR8 infected IL12b-yet40 or WT mice were digested at 37°C with 500μg/mL collagenase D (Sigma; #C-0130), 10μg/mL DNase I (Sigma; #D-4263), and 10mM HEPES in HBSS media. Single cell suspensions were pre-incubated with Fc Block antibody (BD Pharmingen) in PBS/2% FBS for 10 min at 4°C before staining. All antibodies were used at a dilution of 1/200. Cells were stained for 30 min at 4°C, washed, and fixed in 2% paraformaldehyde (Electron Microscopy Sciences) in PBS for 10 min at 4°C. All flow analysis samples were run on a CantoII flow cytometer (BD Biosciences). The resulting data was analyzed using FlowJo software (Treestar).

### Viral titration

BAL fluid, cleared of cells by centrifugation, was used to quantify the amount of infectious virus present in the lungs. Additionally, whole lungs were collected following the BAL procedure and stored at -80°C. At time of virus titer, lungs were thawed, homogenized in 1.0 mL of MEM (minimal essential medium), spun for 5 min at 1000xg to remove cellular debris and the homogenate supernatant used for titers. Virus titers were determined on Madin-Darby Canine Kidney cells (MDCK cells; kindly provided by Dr. Richard Webby at St. Jude Children’s Research Hospital) as previously described [17]. Briefly, serial dilutions of the BAL fluid (10^−1^ to 10^−8^) were incubated on MDCK cells for 1 hour at 37°C followed by a PBS wash. 200μL of infection media (MEM with 1% antibiotics, 1% L-Glutamine, 1% vitamins, 0.1% BSA, 1μg/mL TPCK-trypsin) was added to the cells and incubated at 37°C. After 72 hours, 50μL of the MDCK culture supernatant was mixed with 50μL of 0.5% turkey red blood cells in PBS and the end-point dilution of hemagglutination was scored. TCID_50_ titers were calculated as described [63].

### Histology

For lung histological analysis, female mice were infected with 100 EID_50_ of H5N1 VN/PR8 were sacrificed on day 5 post-infection by Avertin overdose followed by cervical dislocation. Lungs were inflated with 1.0ml of 4% methanol-free paraformaldehyde (Electron Microscopy Sciences, catalog 15710). Lungs were removed and incubated in a 50mL conical tube containing 15mL 4% paraformaldehyde. After 48 hours, lungs were washed for 15 minutes in serial washes of PBS, 30% ethanol, 50% ethanol, and then stored in 70% ethanol at 4°C until processing for paraffin embedding, sectioning, and hematoxylin and eosin (H&E) staining. H&E stained slides were digitized using a Zeiss Axio Scan Z1 Brightfield/Fluorescence Whole Slide Scanner (Zeiss). Areas of consolidation and total lung area were demarcated and measured using Zeiss Zen Lite image analysis software.

### Multiplex cytokine/chemokine quantification

Cytokine and chemokine levels in BAL fluid were measured using a 23-plex-cytokine array according to the manufacturer’s protocol (Bio-Plex Pro Mouse Cytokine 23-plex Group 1; Bio-Rad). The cytokine screen included IL-1α, IL-1β, IL-2, IL-3, IL-4, IL-5, IL-6, IL-9, IL-10, IL-12p40, IL-12p70, IL-13, IL-17, Eotaxin, G-CSF, GM-CSF, IFN-γ, CXCL1, CCL2, CCL3, CCL4, CCL5, and TNF-α. Samples were run in duplicate and raw values averaged for analysis.

### Type I interferon (IFN) bioassay

Biological antiviral activity of type I IFN in BAL fluid were assessed using a cytopathic effect (CPE) assay with L929 cells and the interferon sensitive EMCV (L929 cells and EMCV, Strain K,1x10^8^ PFU/mL; kindly provided by Dr. Michael Diamond at Washington University in St. Louis). BAL fluid was acid-treated with citrate buffer (pH 3.0, 40mM citric acid, 10mM KCl, 125mM NaCl) to inactivate IAV virus; IFN is acid-stable and remains active. Serial dilutions of BAL fluid were made in DMEM/2% FBS and added to L929 cells (3x10^4^ cells/well; plated 8 hours prior) in a 96-well plate. Following incubation for 14 h at 37°C, cells were infected with EMCV diluted in DMEM/2% FBS at a multiplicity of infection (MOI) of 5. Cells were inspected for CPE starting at 10 hours post-infection. An IFN-β standard curve, no IFN, and no EMCV controls were used to assess the time-point of maximal CPE (~10 hours). IFN-mediated protection was assayed using a CellTiter 96 aqueous cell proliferation assay as per the manufacturer's instructions (Promega Corporation, Madison, WI).

### ELISA

Quantification of IL-12p40 concentration (pg/mL) in Fig 3D, Fig 4C and Fig 5A and 5B were performed using the Mouse IL-12/IL-23 p40 allele-specific DuoSet ELISA (R&D Systems; DY499-05) according to manufacturer’s instructions. For S4B Fig, a specific IL-17 ELISA was performed on Nunc Maxisorp plates using anti-IL-17A (clone TC11-18H10.1) as the capture antibody and anti-IL-17A (clone TC11-8H4) as the detection antibody. ELISAs was developed with streptavidin-HRP (BioLegend) and TMB substrate (BioLegend), and absorbance was measured at 450nm and 540nm (background) using a Synergy H1 plate reader (BioTek).

### qRT-PCR

For BMDMs, total cellular RNA was extracted from BMDMs in 200μl of TRK RNA lysis buffer (Omega BioTek) and total RNA was extracted according to the manufacturer’s protocol. RNA samples were treated with DNAse (Turbo DNAse, Ambion) and 200ng of total RNA was reverse transcribed (SuperScript III Reverse Transcriptase, Invitrogen), cDNA was amplified using Taqman reagents on the ABI 7500 Fast PCR detection system. The previously published primer and probe sequences [66] for murine IL-12p40 have the following sequences:

    forward primer 5’-GGAAGCACGGCAGCAGAATA-3’,

    reverse primer 5’-AACTTGAGGGAGAAGTAGGAATGG-3’, and

    probe 5’-CATCATCAAACCAGACCCGCCCAA-3’.

Fluorogenic probes are FAM-labeled at the 5’-end and TAMRA-labeled at the 3’ end. For IL-12p40, to calculate fold change, experimental IL-12p40 Ct values were normalized to the respective β-actin Ct values for the samples. Fold increase relative to mock-treated BMDMs was determined using ΔΔCt calculation.

*Ifi35* mRNA expression in BMDMs and RNA isolated from lung homogenates (using TRIzol; Ambion-Life Technologies) was quantified by qRT-PCR using a SYBR assay and primers that cross a 7.9kB intron and validated using an absolute Ifi35 cDNA plasmid standard curve (BC008158.1). Values were normalized to β-actin Ct values for each respective sample. Fold increase relative to mock-treated BMDMs or uninfected lung sample was determined using ΔΔCt calculation.

    *Ifi35* forward primer 5’-CCATGTCTGTGACCCTGCAA-3’

    *Ifi35* reverse primer 5’-CTCCGGGAGAGCCTGTGC-3’

    β-actin forward primer 5’-GCACAGGGTGCTCCTCAG-3’

    β-actin reverse primer 5’-CTAGGCACCAGGGTGTGATG-3’

### Western blot

For detection of *in vivo* murine IL-12 family members (IL-12p40, IL-23, IL-12p70, and IL-12p80) by fluorescent western blot, cell-free BAL fluid was concentrated 10-fold using a Vivaspin 10K MWCO PES concentrator (Sartorius; VS0101) and subjected to 7.5% PAGE under non-reducing conditions (without 2-mercaptoethanol). Protein was transferred to PVDF membrane for probing with rat anti-mouse IL-12p40 mAb IgG (anti-IL-12p40 polyclonal; clone C17.8; Santa Cruz) overnight in TBST/5% milk at 4°C with agitation. Following a brief wash in TBST/5% milk, the blot was incubated for 2 hours with Alexa Fluor 790 conjugated goat anti-rat (Abcam, ab175786) for imaging and quantification with the LiCor Odyssey imager software. Blots were then stripped and re-probed for IL-17 (anti-IL-17 rat monoclonal; R&D systems, MAB421-SP) as the loading control given that IL-17 levels were not different between WT and *Ifi35*^*-/-*^ mice in two independent assays (23-plex-cytokine array and IL-17 ELISA; see above). IL-12p80 bands normalized to IL-17 expression and quantified relative to the C57Bl/6N IL-12p80 band for each independent experiment.

IL-12p40 detection from *in vitro* BMDM concentrated supernatant or cell lysate was performed similarly however, a donkey-anti-rat HRP secondary antibody was used for chemiluminescent visualization using ECL substrate (Immobilon Western Chemiluminescent HRP Substrate; EMD Millipore) and imaged by Chemidoc imaging system (BioRad). Cell lysate western blots were stripped and re-probed for β-actin (anti-β-actin rabbit polyclonal; Abcam, ab8227) as loading control.

### Bone marrow derived macrophage culture and TLR stimulation

Bone marrow was harvested from 6- to 8-week-old male C57Bl/6N or *Ifi35*^*-/-*^ mice and cultured in macrophage growth media consisting of DMEM, 10% FBS, 10% M-CSF containing CMG-L929 conditioned media, 1% penicillin/streptomycin, 1% L-Glutamine, and 1% sodium pyruvate. Mature, adherent cells were collected on day 7 after harvest and plated in 6-well plates at 5x10^5^ cells/well. Two days later, cells were stimulated with 2.5μg/mL of HMW poly I:C (HMW poly I:C; Invivogen #tlr3-pic), 25ng/mL of R848 (Resiquimod, Invivogen), or 5μg/mL of LPS (List Biologicals; #201). Cell supernatants and cell lysates were collected at the indicated time-points.

### Anti-IL-12p80 antibody and rIL-12p80 administration

For weight loss experiments, 6- to 8-week old female WT C57Bl/6N mice were treated with 100μg of anti-IL-12p80 antibody (Rush University, clone a3-1d) or with Armenian Hamster polyclonal IgG isotype control (Biolegend; #400940) intraperitoneally one day prior to 100 EID_50_ H5N1-VN/PR8 challenge and 3 days following infection. Mice were monitored for weight loss as described above. For BAL experiments, 6- to 8-week old female mice were treated with 100μg of anti-IL-12p80 antibody or with Armenian Hamster polyclonal IgG isotype control (Biolegend; #400940) intraperitoneally one day prior to 100 EID_50_ H5N1-VN/PR8 challenge and sacrificed for BAL at 3 dpi as described above. Additionally, recombinant IL-12p80 (Biolegend; 573102) was intranasally administered to 6- to 8-week old male C57Bl/6N mice one day post-infection with H5N1-VN/PR8. 50ng of IL-12p80 was diluted in 50uL of PBS/0.01% BSA with WT control mice receiving 50uL of PBS/0.01%. Mice were sacrificed for BAL at 3 dpi as described above.

### Statistical information

All *in vivo* data is from at least three independent biological experiments with multiple mice in each group per experiment. Values for individual mice are shown in all graphed data as individual symbols. No blinding was performed during animal experiments. All *in vitro* data is from at least two (IL-12p40 western blots) or three (BMDM stimulation) independent biological experiments with at least two technical replicates per experiment. Statistical differences were calculated using Prism software (GraphPad Prism, San Diego, CA). Kaplan-Meier survival curves were analyzed by the Mantel-Cox Log-rank test. Differences in morbidity were analyzed by Student’s t-test. Littermate experiments and bone marrow chimera data were analyzed by using one-way ANOVA with multiple comparisons correction (Tukey’s comparison correction). Cytokine levels and differences in immune cell subsets were analyzed by Student’s t-test with correction for multiple comparisons Holm-Sidak method. Differences with a *P* value of < 0.05 were defined as statistically significant unless otherwise stated in the figure legend.

## Supporting information

S1 Fig Genotyping of *Ifi35*^-/-^ mice and characterization of immune cells subsets in major organs. **(A)** *Ifi35* gene deletion was verified by genomic PCR. Genotyping was verified with a positive control WT C57Bl/6N (WT) tail DNA and initial *Ifi35*^-/+^ x *Ifi35*^-/+^ breeding pair pups and primers designed by KOMP based on the Velocigene targeting strategy. *Ifi35* deletion was confirmed by the presence of a 210 bp size band, whereas WT manifests as a 105 bp size band and heterozygous mice with both bands. **(B)** WT or *Ifi35*^*-/-*^ BMDMs measured for *Ifi35* mRNA expression following IFN-β and IFN-γ stimulation. **(C)** Total cell number from naïve WT and *Ifi35*^-/-^ thymus, spleen, and lymph node tissues prior to staining for immunophenotyping. **(D,E)** Immunophenotyping of naïve WT and *Ifi35*^-/-^ mice. Lymph node, spleen, and thymus were harvested from WT and *Ifi35*^-/-^ and processed for flow cytometry analysis of **(D)** myeloid Pan DCs (CD11c^+^, MHC-II^+^), monocytes (CD11b^+^, Ly6C^hi^), macrophages (CD64^-^, MerTK^+^, CD11c^-^, F4/80^-^), neutrophils (CD64^-^, CD11c^-^, CD11b^+^, Ly6G^+^) and **(E)** lymphoid CD4^+^ T cells (CD3^+^, NK1.1^-^, CD4^+^), CD8^+^ T cells (CD3^+^, NK1.1^-^, CD8^+^), B cells (CD3^-^, NK1.1^-^, CD19^+^, CD45r^+^), NK cells (NK1.1^+^, CD3^-^), double positive thymocytes (CD4^+^, CD8^+^) and double negative thymocytes (CD4^-^, CD8^-^). Representative graphs from three experiments with WT (n = 6) and *Ifi35*^-/-^ (n = 6) mice. Each symbol represents an individual mouse. Bars represent the mean from at least three different experiments (± SEM). No significant differences were seen in immune subset percentages. n.s., not significant, ***P < 0.0001 using multiple student t-test.
(EPS)

S2 Fig Survival, viral titer in whole lung tissues, and histopathological analysis of whole lungs after IAV infection. **(A)** C57Bl/6N (WT; black) or *Ifi35*^-/-^ (red) mice were intranasally infected with 10^4^ TCID_50_ of highly pathogenic H5N1 (A/Hong Kong/213/2003), or **(B)** 100 EID_50_ of H5N1-Vietnam/PR8 (H5N1-VN/PR8; described in Materials and Methods), or **(C)** 10^4^ TCID_50_ of pandemic H1N1 A/California/04/2009 and monitored for mortality. **(D)** Littermate *Ifi35*^+/+^ (black), *Ifi35*^+/-^ (grey), or *Ifi35*^-/-^ (red) mice were infected with 100 EID_50_ of H5N1-VN/PR8 and monitored for mortality. Survival curves with an imposed 20% weight loss cut-off are shown as dotted lines. Survival curves with a 30% weight loss cut-off are shown as solid lines. n = 18–29 per group. Kaplan-Meier survival curves were analyzed by the Mantel-Cox Log-rank test. *P* values are shown as compared to WT mice. **(E)** Viral titer (log_10_ TCID_50_/mL) determined from homogenized whole lung tissues from WT or *Ifi35*^-/-^ mice at indicated times post-infection with H5N1-VN/PR8. n = 8–14 mice for each time-point from at least three independent experiments. Each symbol represents an individual mouse. Bars represent the mean from at least three different experiments (± SEM). n = 5–13 per group per time point. Assay LOD indicated at 1x10^2^ TCID_50_/mL; dotted line. **(F)** Hematoxylin and eosin staining of whole lung paraffin embedded sections from WT and *Ifi35*^*-/-*^ mice at 5 dpi with H5N1-VN/PR8. Representative lung image shown with black arrows indicating areas of lung consolidation. **(G)** Percent lung involvement as a measure of total consolidated area of the total lung area. Affected areas were demarcated and total area measured for n = 6 mice for each group using Zeiss Lite image analysis software. Each symbol represents an individual value with the mean ± SEM. Open square symbols (open black square and open red square) correspond to the mouse for which representative whole lung H&E images are shown in (F). **(H)** *Ifi35* mRNA expression kinetics in lung homogenate from naïve C57Bl/6 mice or C57Bl/6 mice infected with H5N1 at days 1, 3, 7, and 10 post-infection. n = 3–7 per group from at least two independent experiments. *P < 0.01, **P < 0.001, ***P < 0.0001 using multiple student t-test. n.s., not significant.
(EPS)

S3 Fig Immune cell characterization in bronchoalveolar lavage from wild type and *Ifi35*^-/-^ mice after IAV infection. **(A)** Total number of cells isolated from bronchoalveolar lavage from naïve, infected WT (black), and *Ifi35*^-/-^ (red) mice at indicated times post infection. **(B)** Total number of CD45^+^ alveolar macrophage (CD11c^+^, CD11b^-^, MHC-II^-^, F4/80^+^), neutrophil (PMNs; CD11b^+^, CD11c^-^, Ly6G^+^), monocyte (CD11b^+^, Ly6G^-^, Ly6C^hi^), and dendritic cell (CD11c^+^, CD11b^-^, MHC-II^+^) numbers for naïve mice and H5N1-VN/PR8 infected WT or *Ifi35*^*-/-*^mice at days 1 to 11 in BAL. **(C)** Total number of CD3^+^, CD44^+^ lymphoid CD4^+^, CD8^+^, or γδ-TCR^+^ T cells in BAL of H5N1-VN/PR8 infected WT or *Ifi35*^*-/-*^ mice on days 1 to 11 post infection. n = 8–11 for each group/time point post-infection. n = 3–4 for naïve samples. Each symbol represents an individual mouse. Bars represent the mean from at least three different experiments (± SEM). *P < 0.01, **P < 0.001, ***P < 0.0001 using multiple student t-test. PMN, polymorphonuclear cells; n.d., not detected; n.s., not significant.
(EPS)

S4 Fig IL-12p70, IL-17 cytokine levels, and type I IFN production are not different between H5N1-VN/PR8 infected C57Bl/6N and *Ifi35*^*-/-*^ mice. **(A)** IL-12p70 concentrations (pg/mL) in bronchoalveolar lavage fluid (BALF) from WT (black circle) and *Ifi35*^-/-^ mice (red circle) at indicated time-points post infection with H5N1-VN/PR8 as measured by Bio-Plex Pro Mouse Cytokine 23-Plex assay. **(B)** IL-17 concentrations in BALF from WT or *Ifi35*^*-/-*^ mice at 3 dpi and 5 dpi as measured by ELISA. T cell supernatant was used as a positive control for the assay. Samples were run in duplicate and selected from two independent experiments. Dotted line (red) indicates limit of detection (LOD) based on internal standard curve values. **(C)** Type I IFN in BALF from WT or *Ifi35*^-/-^ mice infected with H5N1-VN/PR8, measured using an IFN Bioassay. Each symbol represents an individual mouse. Bars represent the mean from at least two different experiments (± SEM) and analyzed using multiple student t-test. n.s., not significant.
(EPS)

S5 Fig Whole lung flow cytometry of H5N1-VN/PR8 infected IL-12p40-IRES-eYFP reporter mice. **(A)** Representative whole lung flow cytometry plots to depict the gating strategy for IL12b-yet40 homozygous mice (IL-12p40-IRES-eYFP reporter) at 3 or 5 dpi with H5N1-VN/PR8. Numbers on gates represent frequency of unstained live, single cells then gated on FITC reporter signal only or **(B)** Frequencies of anti-CD45 stained live, single, FITC^+^ cells that are then subjected to CD45 gating. **(C)** Gating strategy following live, single, FITC^+^, CD45+ cell population selection from IL12b-yet40 homozygous mice at 5 dpi with H5N1-VN/PR8. Graph depicts mean (± SEM) frequency of monocytes (Ly6C+, Cd11b+) or dendritic cells (MHC-II+, Cd11c+) as a percentage of the total FITC+ population.
(EPS)

S6 Fig Source gel images for BMDM and BAL IL-12p40. **(A)** Source gel images for Fig 5 showing Chemiluminescent image overlaid with White Epi image (performed on ImageJ) for BMDM supernatant following HMW poly I:C treatment, **(B)** BMDM supernatant following LPS and R848 treatment, and **(C)** whole cell lysate from BMDMs treated with HMW poly I:C. Low (left) and high (right) exposure time images are shown with outlined boxes to indicate portions used for main figure. Whole cell lysate blot was stripped and re-probed for β-actin as a loading control. **(D)** Source gel images for Fig 6 showing fluorescent image scanned using the LiCor Odyssey Imager. Bronchoalveolar lavage fluid from C57Bl/6N or *Ifi35*^*-/-*^ mice on 5 dpi following H5N1-VN/PR8 infection were concentrated and run on a 7.5% non-reducing PAGE gel as outlined in the Methods. Low (left) and high (right) exposure time images are shown with outlined boxes to indicate portions used for main figure. The same blot was then re-probed for IL-17 as a loading control. Refer to Key Resources Table (S2 Table) for all antibodies used. **(E)** Quantification of percent IL-12p40 monomer and percent IL-12p80 of total IL-12p40 signal. Band signal intensities for IL-12p40 monomer and IL-12p80 were normalized to IL-17. Total IL-12p40 signal was calculated as the sum of IL-12p40 monomer and IL-12p80 normalized signal. IL-12p80 normalized signal was halved to reflect two IL-12p40 antibody binding sites. **(F)** Anti-IL-12p80 or Hamster IgG Isotype treated mice were infected with 100 EID_50_ of H5N1-VN/PR8 and monitored for mortality. Survival curves with an imposed 20% weight loss cut-off are shown as dotted lines. Survival curves with a 30% weight loss cut-off are shown as solid lines. n = 9–16 per group. Kaplan-Meier survival curves were analyzed by the Mantel-Cox Log-rank test.
(EPS)

S1 Table Cytokine and chemokine profiles of C57Bl/6N and *Ifi35*^*-/-*^ mice. Mean ± SEM values for all 23 cytokines assayed in C57Bl/6N and *Ifi35*^*-/-*^ mice at 1, 2, 3, 5, 8 and 11 dpi. Values are from three independent experiments with total of n = 9–19 mice per group. *P < 0.01, **P < 0.001, ***P < 0.0001 using multiple student t-test.
(PDF)

S2 Table Key resources used in experiments, data processing, and analysis. (PDF)
