# Supplementary material for: Interferon induced protein 35 exacerbates H5N1 influenza disease through the expression of IL-12p40 homodimer
Source: PLoS Pathog. 2018 Apr 26;14(4):e1007001. doi: 10.1371/journal.ppat.1007001 (PMC5940246; doi:10.1371/journal.ppat.1007001)
Supplement: S1 Table — Mean ± SEM values for all 23 cytokines assayed in C57Bl/6N and Ifi35-/- mice at 1, 2, 3, 5, 8 and 11 dpi. Values are from three independent experiments with total of n = 9–19 mice per group. *P < 0.01, **P < 0.001, ***P < 0.0001 using multiple student t-test. (PDF) [file ppat.1007001.s007.pdf]

S1 Table. Cytokine and chemokine profiles of C57Bl/6N and *Ifi35*$^{-/-}$ mice. Mean ± SEM values for all 23 cytokines assayed in C57Bl/6N and *Ifi35*$^{-/-}$ mice at 1, 2,3, and 5 dpi following infection with 100EID$_{50}$ of H5N1-VN/PR8. Values are from three independent experiments with total of n = 9-19 mice per group. *$P < 0.01$, **$P < 0.001$, ***$P < 0.0001$ using multiple student t-test. n.d. = not detectable

	**Cytokine/chemokine concentration (pg/mL) in bronchoalveolar lavage fluid following 100 EID$_{50}$ H5N1-IAV challenge:**					
	**Day 1**		**Day 2**		**Day 3**	
	**C57Bl/6N** (n = 9) mean ± SEM	***Ifi35*$^{-/-}$** (n = 15) mean ± SEM	**C57Bl/6N** (n = 14) mean ± SEM	***Ifi35*$^{-/-}$** (n = 19) mean ± SEM	**C57Bl/6N** (n = 10) mean ± SEM	***Ifi35*$^{-/-}$** (n = 14) mean ± SEM
**IL-1a**	5.13 ± 1.48	4.05 ± 0.81	4.15 ± 0.91	3.92 ± 0.77	4.30 ± 1.21	4.67 ± 0.70
**IL-1b**	58.61 ± 11.81	60.10 ± 14.57	65.00 ± 7.97	8.74 ± 6.38	577.00 ± 3.53	74.14 ± 3.87
**IL-2**	3.51 ± 0.53	2.07 ± 0.64	0.96 ± 0.42	n.d.	4.04 ± 0.56	4.28 ± 0.63
**IL-3**	2.43 ± 0.21	2.14 ± 0.37	1.73 ± 0.25	1.42 ± 0.26	1.77 ± 0.07	1.72 ± 0.17
**IL-4**	n.d.	n.d.	n.d.	n.d.	n.d.	n.d.
**IL-5**	1.69 ± 0.74	1.14 ± 0.59	1.62 ± 0.48	0.81 ± 0.22	8.27 ± 0.99	9.58 ± 1.17
**IL-6**	3.49 ± 0.47	3.17 ± 1.12	62.57 ± 7.72	40.75 ± 4.56	200.52 ± 22.85	149.99 ± 15.81
**IL-9**	54.95 ± 11.53	35.15 ± 12.75	35.09 ± 9.28	21.70 ± 7.45	10.53 ± 7.02	11.67 ± 8.02
**IL-10**	3.84 ± 2.58	3.52 ± 2.37	5.34 ± 2.01	3.69 ± 1.18	27.01 ± 1.53	23.73 ± 1.51
**IL-12(p40)**	44.04 ± 3.55	19.07**** ± 1.11	89.01 ± 6.93	41.57**** ± 3.43	267.20 ± 5.51	154.35*** ± 16.84
**IL-12(p70)**	17.42 ± 1.39	13.25 ± 3.10	14.12 ± 1.44	9.05 ± 1.41	29.26 ± 1.83	27.80 ± 1.43
**IL-13**	59.08 ± 6.74	57.65 ± 12.26	41.44 ± 7.25	38.27 ± 6.32	20.29 ± 6.93	15.22 ± 6.47
**IL-17**	13.87 ± 1.04	12.75 ± 0.89	11.77 ± 0.53	11.78 ± 0.43	13.37 ± 0.88	12.48 ± 0.64
**Eotaxin**	87.29 ± 26.36	80.78 ± 20.03	58.48 ± 18.85	83.59 ± 19.34	225.56 ± 13.39	220.72 ± 8.52
**G-CSF**	8.38 ± 1.65	5.27 ± 1.11	42.5 ±1 3.65	34.62 ± 4.72	137.23 ± 18.77	132.84 ± 16.16
**GM-CSF**	35.75 ± 10.53	37.29 ± 9.09	43.50 ± 8.09	36.26 ± 6.12	89.55 ± 3.14	82.96 ± 1.42
**IFN-γ**	1.47 ± 0.38	1.74 ± 0.64	4.93 ± 2.03	4.30 ± 0.78	8.58 ± 0.76	12.98 ± 2.55
**KC**	23.18 ± 3.28	18.06 ± 2.02	84.27 ± 10.65	44.47** ± 3.41	345.60 ± 12.52	140.92**** ± 11.92
**MCP-1**	45.39 ± 8.51	41.30 ± 10.95	86.76 ± 9.64	58.27* ± 5.50	245.06 ± 16.89	223.91 ± 29.95
**MIP-1a**	12.98 ± 3.70	9.21 ± 1.71	26.42 ± 4.88	17.66 ± 1.94	21.43 ± 2.02	17.32 ± 1.72
**MIP-1b**	4.77 ± 0.61	6.88 ± 3.39	51.45 ± 17.36	29.57 ± 4.84	27.85 ± 3.65	33.05 ± 4.45
**RANTES**	3.50 ± 0.58	2.63 ± 0.79	24.79 ± 3.55	16.33 ± 1.40	36.14 ± 4.85	31.40 ± 4.76
**TNF-a**	51.04 ± 9.62	47.88 ± 6.53	74.37 ± 19.48	54.47 ± 12.46	105.41 ± 27.20	78.96 ± 17.06

	**Cytokine/chemokine concentration (pg/mL) in bronchoalveolar lavage fluid following 100 EID$_{50}$ H5N1-IAV challenge:**					
	**Day 5**		**Day 8**		**Day 11**	
	**C57Bl/6N** (n = 12) mean ± SEM	**C57Bl/6N** (n = 12) mean ± SEM	**C57Bl/6N** (n = 11) mean ± SEM	***Ifi35$^{-/-}$*** (n = 10) mean ± SEM	**C57Bl/6N** (n = 7) mean ± SEM	***Ifi35$^{-/-}$*** (n = 6) mean ± SEM
**IL-1a**	5.99 ± 0.90	5.99 ± 0.90	1.15 ± 0.52	1.27 ± 0.47	n.d.	n.d.
**IL-1b**	106.36 ± 5.60	106.36 ± 5.60	53.58 ± 6.72	72.13 ± 3.40	40.29 ± 12.08	46.19 ± 2.67
**IL-2**	5.81 ± 0.47	5.81 ± 0.47	4.67 ± 0.67	5.63 ± 1.08	n.d.	0.49 ± 0.49
**IL-3**	2.98 ± 0.87	2.98 ± 0.87	7.53 ± 1.8	6.30 ± 1.38	0.61 ± 0.41	n.d.
**IL-4**	n.d.	n.d.	n.d.	n.d.	n.d.	n.d.
**IL-5**	34.72 ± 3.81	34.72 ± 3.81	24.31 ± 4.25	40.98 ± 9.67	5.37 ± 1.51	2.86 ± 1.14
**IL-6**	274.17 ± 18.68	274.17 ± 18.68	105.63 ± 29.03	203.93 ± 48.16	10.86 ± 3.28	5.91 ± 2.99
**IL-9**	n.d.	n.d.	6.35 ± 6.35	n.d.	n.d.	n.d.
**IL-10**	51.15 ± 2.25	51.15 ± 2.25	64.37 ± 13.41	59.12 ± 13.67	14.99 ± 3.61	11.29 ± 1.57
**IL-12(p40)**	714.71 ± 29.22	714.71 ± 29.22	170.78 ± 13.38	141.42 ± 20.63	180.43 ± 37.65	102.54 ± 18.05
**IL-12(p70)**	36.02 ± 2.51	36.02 ± 2.51	28.23 ± 6.41	29.16 ± 4.27	4.79 ± 0.88	3.25 ± 1.10
**IL-13**	28.40 ± 5.32	28.40 ± 5.32	13.59 ± 6.04	8.54 ± 4.77	2.02 ± 2.02	n.d.
**IL-17**	13.61 ± 0.84	13.61 ± 0.84	1.53 ± 0.87	1.17 ± 0.60	n.d.	n.d.
**Eotaxin**	119.89 ± 36.63	119.89 ± 36.63	211.38 ± 25.67	175.84 ± 24.22	65.99 ± 23.61	62.22 ± 27.88
**G-CSF**	785.60 ± 68.66	785.60 ± 68.66	280.06 ± 47.31	250.44 ± 42.27	28.40 ± 11.88	12.14 ± 5.55
**GM-CSF**	62.60 ± 3.66	62.60 ± 3.66	20.20 ± 7.17	39.78 ± 5.85	17.02 ± 6.63	15.25 ± 9.70
**IFN-γ**	10.85 ± 0.69	10.85 ± 0.69	257.06 ± 74.49	159.67 ± 68.09	2.17 ± 0.61	0.60 ± 0.38
**KC**	469.09 ± 37.84	469.09 ± 37.84	53.26 ± 6.52	71.65 ± 10.87	35.37 ± 7.49	23.73 ± 8.48
**MCP-1**	1180.04 ± 208.09	1180.04 ± 208.09	535.98 ± 81.19	385.63 ± 72.87	71.95 ± 35.23	4.74 ± 4.74
**MIP-1a**	50.81 ± 4.05	50.81 ± 4.05	18.97 ± 3.55	19.03 ± 2.54	3.62 ± 1.14	0.50 ± 0.50
**MIP-1b**	120.52 ± 11.47	120.52 ± 11.47	48.35 ± 6.08	41.90 ± 6.10	9.27 ± 3.01	2.12 ± 1.12
**RANTES**	82.90 ± 8.65	82.90 ± 8.65	34.63 ± 13.28	20.68 ± 1.92	12.56 ± 2.18	6.53 ± 1.63
**TNF-a**	224.80 ± 32.70	224.80 ± 32.70	142.00 ± 18.26	118.60 ± 25.23	64.76 ± 15.98	53.51 ± 15.34
