# Supplementary material for: Interferon induced protein 35 exacerbates H5N1 influenza disease through the expression of IL-12p40 homodimer
Source: PLoS Pathog. 2018 Apr 26;14(4):e1007001. doi: 10.1371/journal.ppat.1007001 (PMC5940246; doi:10.1371/journal.ppat.1007001)
Supplement: S2 Table — (PDF) [file ppat.1007001.s008.pdf]

S2 Table: Key resources used in experiments, data processing, and analysis.

REAGENT or RESOURCE	SOURCE	IDENTIFIER
**Antibodies (Main Figures)**		
PE anti-Ly6G; 1A8	BioLegend	127608
PerCP/Cy5.5 anti-Ly6C; HK1.4	BioLegend	128012
PE/Cy7 anti-CD11b; M1/70	Tonbo	60-0112
APC anti-F4/80; BM8.1	Tonbo	20-4801
APC/Cy7 anti-CD11c; N418	Tonbo	25-0114
BV510 anti-CD45; 30-F11	BD Pharmingen	563891
Pacific Blue anti-I-a/I-e; M5/114.15.2	BioLegend	107620
FITC anti-CD3e; 145-2C11	BioLegend	100306
PerCP/Cy5.5 anti-CD8α; 53-6.7	Tonbo	65-0081
PE/Cy7 anti-CD4; RM4-5	BioLegend	100528
APC anti-TCR $\gamma\delta$; GL3	BioLegend	118116
APC-Cy7 anti-CD45; 30-F11	BD Pharmingen	557659
V500 anti-B220; RA3-6B2	BD Pharmingen	561226
Pacific Blue anti-CD44; IM7	BioLegend	103020
Hamster anti-mouse IL-12p80; a3-1d	Kalipada Pahan (Rush University)	a3-1d
Rat anti-mouse IL-12p40; C17.8	Santa Cruz Biotechnology	sc-57258
Rat anti-mouse IL-17/IL-17A; 50104	R&D Systems	MAB421
Alexa Fluor 790 goat anti-rat	Abcam	ab175786
**Additional antibodies in supplementary figures**		
PE anti-CD45R/B220; RA3-6B2	BD Pharmingen	553090
PE/Cy7 anti-CD8α	BD Pharmingen	552877
APC anti-NK1.1; PK136	BioLegend	108709
APC/eFluor780 anti-CD11c; N418	eBioscience	47-0114-82
Pacific Blue anti-CD19; 6D5	BioLegend	115523
BV605 anti-CD4; RM4-5	BioLegend	92294
PerCP/Cy5.5 anti-I-a/I-e	BioLegend	107626
FITC anti-F4/80; BM8	BioLegend	123108
PE anti-Ly6C; HK1.4	BioLegend	128007
PE/Cy7 anti-Ly6G; 1A8	BioLegend	127618
APC anti-CD64; X54-5/7.1	BioLegend	139306
BV421 anti-CD11b; M1/70	BioLegend	101235
Biotinylated anti-Mouse MerTK	R&D Systems	BAF591
Qdot-605 Streptavidin	Invitrogen	Q10101MP
**Virus Strains**		
H5N1 A/Hong Kong/213/2003	Boon Lab	n/a
A/Vietnam/1203/2004 + A/Puerto Rico/8/1934 (H5N1-VN/PR8)	Boon Lab	n/a
pH1N1 A/California/04/2009 influenza virus	Boon Lab	n/a
**Chemicals**		
Avertin (2,2,2-tribromoethanol)	Sigma-Aldrich	T48402
Busulfan	Alfa Aesar	J61348
High Molecular Weight poly I:C	Invivogen	tlr3-pic
LPS	List Biologicals	204
Resiquimod (R848)	Invivogen	tlrl-r848

Recombinant mouse IL-12p40 homodimer	Biolegend	573102
**Critical Commercial Assays**		
Bio-Plex Pro Mouse cytokine -23-plex	Bio-Rad	M60009RDPD
Mouse IL-12/IL-23 p40 allele-specific DuoSet ELISA	R&D Systems	DY499
**Experimental Models: Organisms/Strains**		
Mouse: C57BL/6N	Boon Lab	Bred in-house
Mouse: *Ifi35*$^{-/-}$ (*Ifi35*^{tm1}(KOMP)Vlcg)	Boon Lab	Bred in-house
Mouse: IL12b-yet40 (p40-IRES-eYFP)	Jackson Laboratories	006412
Mouse: B6-Ly5.1/Cr (C57Bl/6 CD45.1)	Charles River	564
**Software and Algorithms**		
Statistics: Prism 7	Graphpad	n/a
Flow cytometry analysis: FlowJo software	FlowJo, LLC	n/a
